# Perceptions of symptoms and expectations of advanced therapy for Parkinson’s disease: *preliminary report of a Patient-Reported Outcome tool for Advanced Parkinson’s disease (PRO-APD)*

**DOI:** 10.1186/1477-7525-12-11

**Published:** 2014-01-24

**Authors:** Prashanth Reddy, Pablo Martinez-Martin, Richard G Brown, Kallol Ray Chaudhuri, Jean-Pierre Lin, Richard Selway, Ian Forgacs, Keyoumars Ashkan, Michael Samuel

**Affiliations:** 1 Department of Neurology, King’s College Hospital, London, UK; 2 Research Unit, Alzheimer Center Reina Sofia Foundation and CIBERNED, Carlos III Institute of Health, Madrid, Spain; 3 Department of Psychology, Institute of Psychiatry, King’s College London, London, UK; 4 Department of Pediatrics, Evelina Children’s Hospital, St Thomas Hospital, London, UK; 5 Department of Neurosurgery, King’s College Hospital, London, UK; 6 Department of Gastroenterology, King’s College Hospital, London, UK; 7 Department of Neurology, East Kent Hospitals NHS Foundation Trust, Ashford, UK; 8 National Parkinson Foundation Centre of Excellence, King’s College Hospital, King’s Health Partners, London, UK; 9 Research Fellow in Movement Disorders, Department of Neurology, King’s College Hospital, Denmark Hill SE5 9RS London, UK

## Abstract

**Background:**

What do patients expect from a treatment? A patient-centred approach to treatment is becoming necessary given the choices for invasive treatments for Parkinson’s disease. Patient’s perceptions of severity and expectations from complex therapies have not been studied. We describe the rationale and concept of developing a Patient-Reported Outcome (PRO) tool to assess perceptions of symptom severity and expectations of therapy. We report preliminary findings from use of the tool, association with clinical factors, and illustrate the potential use in individual patients awaiting therapy.

**Methods:**

Patient symptoms were grouped into four domains, with 8 motor, 7 non-motor, 7 psychological and 4 social questions. For each question, symptom severity was rated on a Likert scale scoring from 0 (no problem) to 7 (perceived as a severe problem). Similarly, the expectation for each symptom to change after therapy was rated on a Likert scale: score −3 (expected to be very much worse) to + 3 (expected to be very much improved).

**Results:**

22 consecutive patients, routinely planned to receive one of Deep Brain Stimulation/Intrajejunal Levodopa Infusion/Apomorphine Infusion therapies, were recruited: 13 male, mean (+/−sd) age: 65.6 (+/−9.5) years, mean (+/−sd) disease duration: 14.3 (+/−5.7) years. Subjective severity scores are reported as mean (+/−sd) / maximum possible score: (i) motor 23.5 (+/−7.5) / 56, (ii) non-motor 15.5 (+/−5.6) / 49, (iii) cognitive - psychological 12.4 (+/−5.8) / 49, (iv) social 9.3 (+/−4.1) / 28. Expectation of change (improvement) scores are reported as mean (+/−sd) / maximum possible score of: (i) motor 14.0 (+/−5.6) / 24, (ii) non-motor 8.5 (+/−4.1) / 21, (iii) cognitive - psychological 7.4 (+/−4.4)/ 21, and (iv) social 5.5 (+/−2.8) / 12. For each domain, Spearman correlation coefficient showed significant associations between severity and expectation within-domain.

**Conclusion:**

This tool (PRO-APD) provides a description of perceived problem severity and expectation of treatments encompassing a holistic patient-driven view of care. PD patients about to receive complex therapy have moderately high perception of symptom load in multiple domains, and expect substantial improvements in multiple domains. These preliminary findings may be useful in documenting multi-domain symptoms, as well as counseling patients to help them reach realistic expectations and reduce potential dissatisfaction following therapy.

## Introduction

Parkinson’s disease (PD) is a progressive neurodegenerative disorder affecting a wide range of motor and non-motor functions, and leading to marked disability in its later stages. At a population level, subjective health status (quality of life) is associated with both motor and non-motor symptoms (NMS) [1-3]. However, for individual patients, the perceived significance of different motor and non-motor symptoms is likely to be influenced by the extent to which they interfere with particular aspects of their life, some of which will be specific to the individual (e.g. in relation to occupation and recreation).

Once a treatment is suggested, the question of “how do we know if that treatment works?” remains a complex one. The ability of treatment to manage individually important symptoms may influence a patient’s preferences for and expectations of treatment, for example a watchmaker affected by tremor. Particularly pertinence applies when using complex, invasive, expensive therapies, such as deep brain stimulation (DBS), Intrajejunal Levodopa Infusion (IJL) and subcutaneous Apomorphine (Apo) infusions, because these can offer marked benefits for some but not all symptoms of PD, but require high levels of expertise, high costs and demand significant time requirements from health care providers [4].

Understanding the individual patient’s perceptions of the importance of specific symptoms and expectations of treatment is important clinically for a number of reasons. Firstly, where a choice of treatments is available it may be possible to choose or tailor the treatment better to suit the patient’s requirements. For example, the optimal dose of dopaminergic stimulation or DBS parameters for motor control may not necessarily be optimal for cognition or other NMS. Secondly, it will help patients and those treating them make better-informed decisions amongst treatment options where those treatments vary in how well they deal with specific problems. Thirdly, identifying possibly unrealistic expectations may be useful in preparing patients for the most likely clinical outcome and minimize adverse emotional reactions following treatment. Ultimately, the patient’s perception of the ability of a treatment to improve the personally relevant (patient – centred) aspects of their condition is the primary indicator of that treatment’s success.

Patient-Reported Outcomes (PRO) were defined nearly a decade ago by the US Food and Drug Administration (FDA) as “a patient’s report of a health condition and its treatment” [5]. PRO are subjective assessments by the patient of any aspect of health, e.g. symptoms, functional status, psychological well-being, quality of life, preferences, perceptions, and satisfaction with care [6]. Although there are PRO scales (PDQ-8 and PDQ-39) used in Parkinson’s disease, none to date capture patient perception and expectation from advanced treatment. One published study has explored patient perceptions and expectations to treatment in PD, focusing only on the oral therapies but not the advanced therapies in PD [7]. The present study sought to assess patient perceptions of the severity and importance of a range of symptoms and their personal impact, and their expectation of improvement from the proposed therapy for those symptoms. This preliminary pilot work assessed the practicality of application of such a tool. Secondly we wished to investigate correlations between symptom severity and expectation of change before therapy, as this has not previously been explored for these therapies in PD.

## Methods

The study was approved by the Regional ethics committee of South East London: ref number: 10/H0808/46. The funding source was a grant from King’s Health Partners, obtained through open competition.

A local PRO scale was designed and titled provisionally the Patient Reported Outcomes in Advanced Parkinson’s disease (PRO-APD). The scale was designed taking into consideration our experience with motor, non-motor, social and quality of life issues in Parkinson’s disease. The questions were designed to encompass the spectrum of problems/issues encountered by Parkinson’s disease patients. We have a large local and regional clinic at our hospital and the central themes of the questions was based on our clinical impression, coupled with currently available contemporary views from the literature. In addition, a group of patients was consulted with regards to the content and individual questions before the pilot version of the scale reported here was finalized. There were 8 motor, 7 non-motor, 7 cognitive/psychological and 4 social questions, giving a total of 26 questions (Appendix 1).

Within each domain, the individual symptom severity was first rated by the patient on a visual Likert scale from 0 (“I do not have the problem”) to 7 (“I have a very severe problem”). Next, expectations of therapy for each item were rated on a second Likert scale. A bipolar scale was used with 7 points from −3 (expected to be very much worse) to +3 (expected to be very much improved), with 0 as expected to show no change post-therapy. The scale was administered on paper to the participants with the investigator present to help explain and answer any questions.

Additionally, participants were assessed on a range of other measures, the new Unified Parkinson’s Disease Rating Scale (MDS-UPDRS) [8], Non-motor Symptoms Scale (NMSS) [9], Parkinson’s Disease Questionnaire (8-item) (PDQ-8) [10], Hospital Anxiety and Depression Scale (HADS) [11] and Adenbrooke’s Cognitive Examination Scale (Revised) (ACE-R) [12]. These scales are widely used and validated for use in PD for their selected symptoms.

Participants with PD were included in the study if they had already been selected to receive treatment with one of DBS or IJL or Apo by their clinical teams as part of their routine care, at hospital-based clinical neurology settings at King’s College Hospital NHS Foundation Trust, London UK. Separate informed consent was obtained for entry into the PRO-APD study. The PRO-APD severity and expectation scales were applied following the clinical decision to proceed but before the delivery of that therapy. In our institution, this waiting time can range from 6 weeks to 6 months.

### Data analysis and statistics

For each patient, items within each domain were summed to give a domain score. The maximum possible scores for severity domains were: motor 56, non-motor 49, psychological, 49, social 28. A total score was also calculated to quantify the patient’s overall perceptions of their PD-related problems. Whole group domain and total means were then calculated.

For the expectation components, the ranges of possible scores for change were motor −24 to +24, non-motor −21 to +21, cognitive/psychological −21 to +21, social −12 to +12. Negative values indicated that a patient was expecting a worsening of symptoms after therapy. In practice, no patient expected worsening on any problem. All individual expectation scores and domain scores therefore indicated either anticipations of no change or improvement. For each patient, the scores for expectations of improvement from each question in a domain were summed to give domain scores of expectations of improvement. Whole group domain and total means were then calculated. We also calculated an overall mean total expectation score for improvement, combining all domains.

Because of the different numbers of items in each domain, standardized scores were also calculated expressed as a percentage of the maximum score possible for each domain, for both severity and expectation.

Finally, we investigated total and domain-specific associations between severity and expectation. The Shapiro-Wilk W-test showed that data of interest/variables were compatible with a normal distribution. To assess associations, the Spearman rank correlation coefficient was used due to the small sample size. Correction for multiple comparisons was not carried out, as this was a pilot exploratory study.

## Results

A total of 22 consecutive patients were recruited for the study (8 patients for STN DBS, 11 patients selected for Apo, 3 patients for IJL) of which 13 were male and 9 female. The demographics are shown in Table 1. Table 2 shows the mean scores for each domain for severity and for expectation, along with total scores. Severity scores for the motor domain were rated highest and cognitive/psychological the lowest. The expectations from therapy showed the same pattern across the sample.

**Table 1:** Patient demographics, motor and non-motor severity, quality of life, mood and cognition (N = 22)

**Variable**	**Mean (SD)**	**Range**
Age (years)	65.6 (9.5)	49 – 80
Duration of disease (years)	14.3 (5.7)	5 – 23
MDS-UPDRS-3	51.7 (15.8)	15 – 83
MDS-UPDRS-4	8.6 (6.4)	0 – 20
NMSS total	74.9 (28.9)	39 – 140
PDQ-8 total	37.3 (16.3)	12.5 – 65.6
HADS total	16.3 (6.6)	5 – 28
ACE-R total	90.1 (7.6)	73 – 100

*Abbreviations*: (*MDS-UPDRS* Revised Unified Parkinson’s Disease Rating Scale, *NMSS* Non-Motor Symptoms Scale, *PDQ-8* Parkinson’s disease quality of life scale, *HADS* Hospital Anxiety and Depression Scale, *ACE-R* Addenbrooke’s Cognitive Examination -Revised).

**Table 2:** Domain and total PRO-APD scores of problem severity and expectation of change (N = 22)

**Variable**	**Mean (SD)**	**Range**	**Mean (% maximum) (SD)**	**Correlation of severity with expectation**	**P**
Motor severity (max 56)	23.5 (7.5)	5 – 36	43.4% (13.9)	0.79	<0.001
Motor expectation of improvement (max 24)	14.0 (5.6)	1 – 21	58.3% (22.9)		
NMS severity (max 49)	15.5 (5.6)	6 – 31	31.5% (11.2)	0.72	<0.001
NMS expectation of improvement (max 21)	8.5 (4.1)	2 – 16	40.3% (19.1)		
Cognitive/psychological severity (max49).	12.4 (5.8)	0 – 24	25.2% (11.1)	0.68	<0.001
Cognitive/psychological expectation of improvement (max 21)	7.4 (4.4)	0 – 16	35.3% (20.8)		
Social severity (max 28)	9.3 (4.1)	3 – 16	33.1% (14.6)	0.62	0.002
Social expectation of improvement (max 12)	5.5 (2.8)	0 – 9	45.8% (23.1)		
Total severity (max 182)	60.5 (16.7)	31 – 105	33.3% (9.4)	0.70	<0.001
Total expectation of improvement (max 78)	35.5 (14.1)	12 – 59	45.3% (17.6)		

NMS = non-motor symptoms, SD = standard deviation.

**Figure 1:**
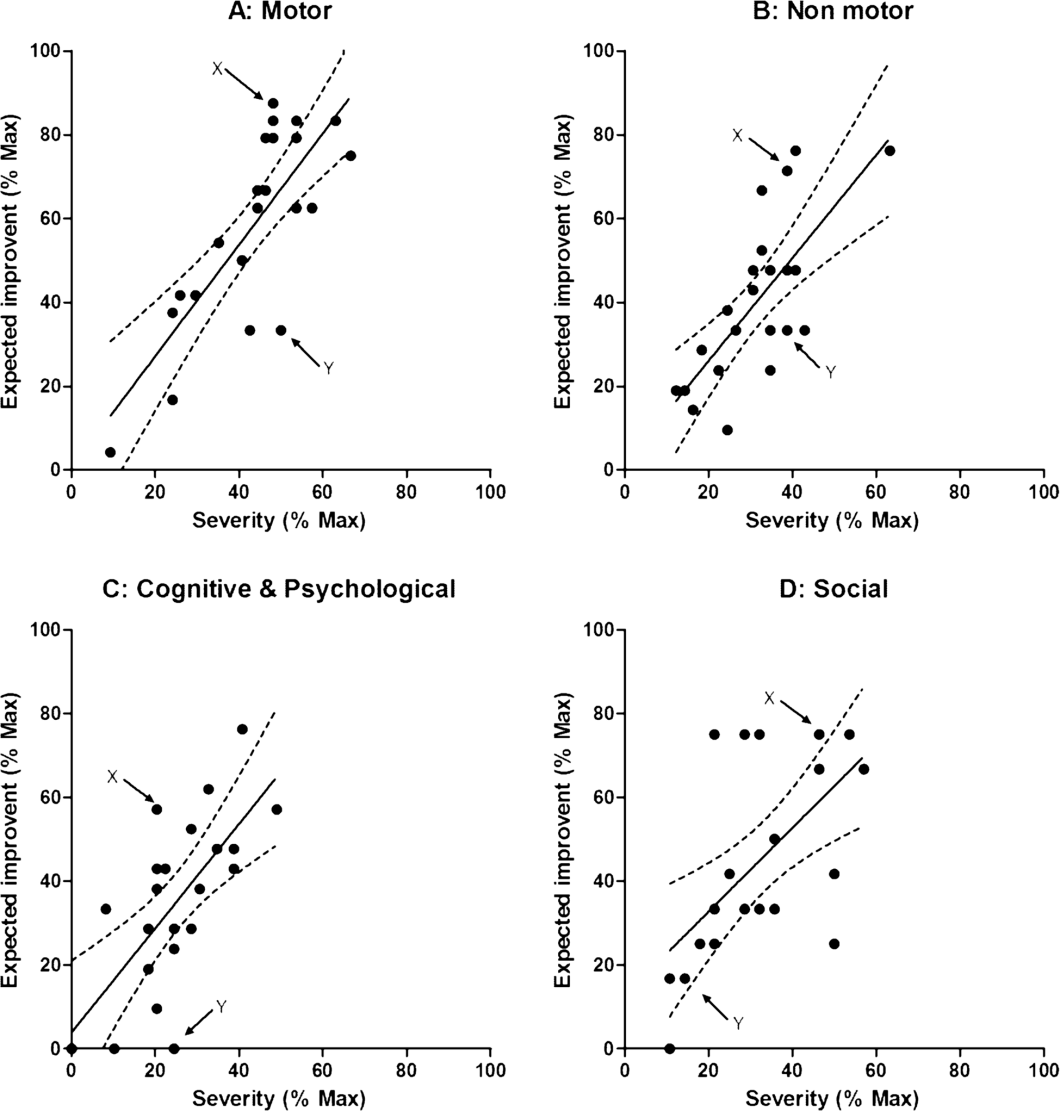
**Scatterplots showing relation between perceived severity and expected improvement for each of the 4 domains (solid line represents the regression line and the dotted line the 95% confidence interval.** Arrows indicate case examples X and Y). For the motor, non-motor and cognitive/psychological domains, the severities in patients X and Y are comparable but expectations differ.

**Figure 2:**
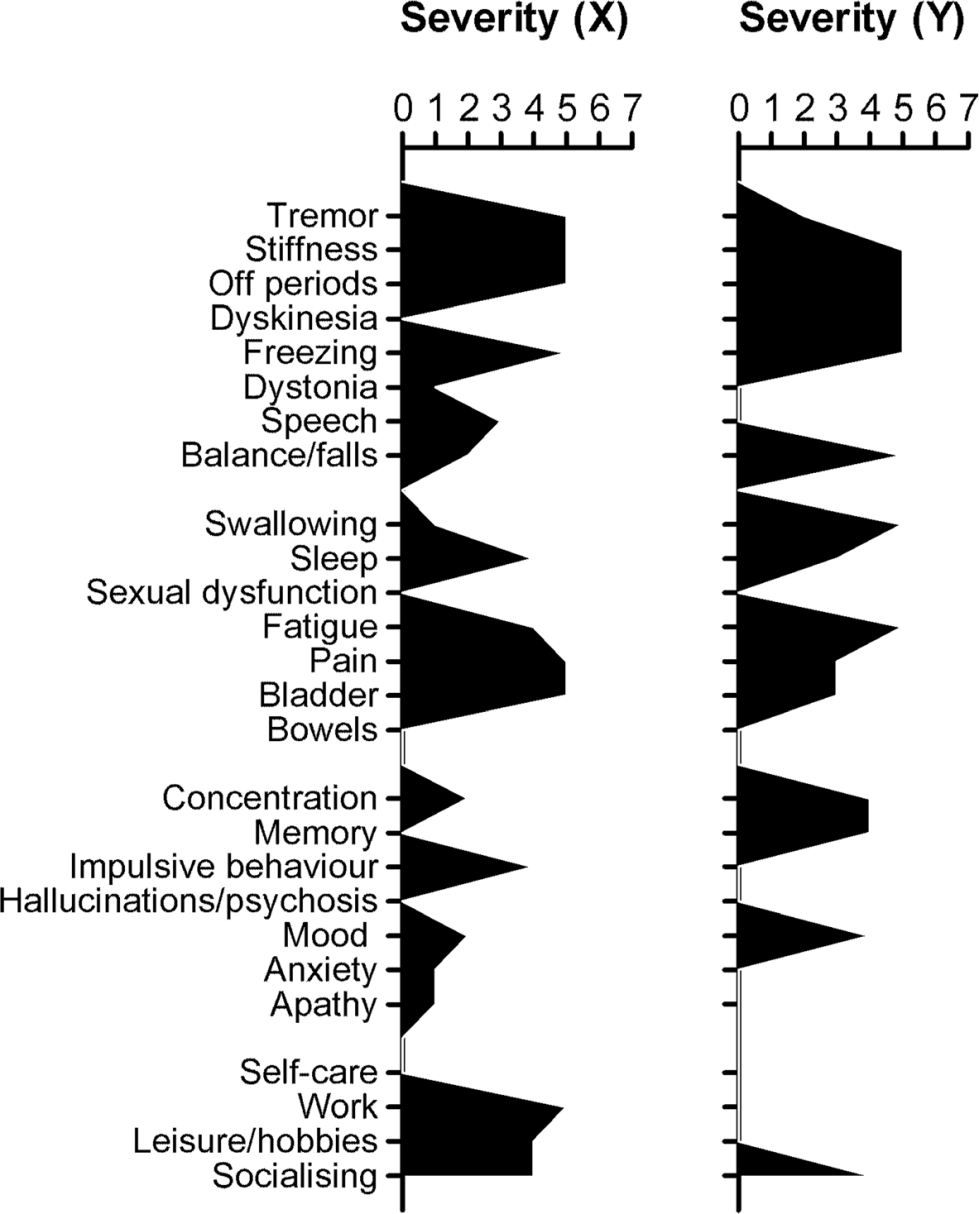
Profiles of perceived severity for illustrative cases X and Y (gaps indicate zero ratings), showing broadly similar severities across individual symptom profiles.

**Figure 3:**
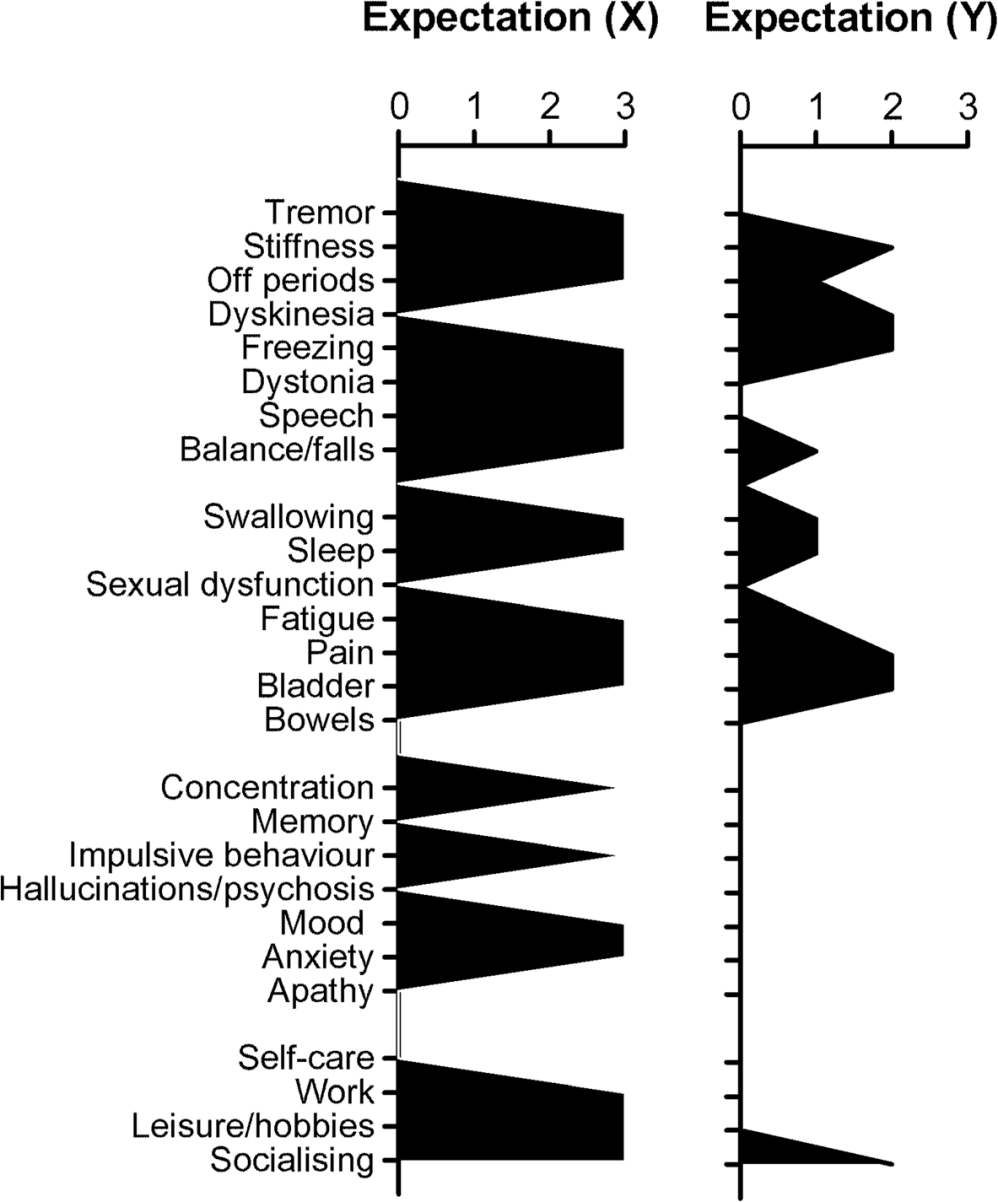
Profiles of expectations of improvement ratings for illustrative cases X and Y (gaps indicate zero ratings) showing clearly different profiles of expectation following treatment.

Within domains, significant associations were found between perceived severity and expectations for therapy in each domain, with higher severity associated with higher expected improvement (Table 2). Although significantly associated in the group as a whole, there was evidence of variability at the individual level between ratings of severity and expectation. The scatterplots for each domain are shown in Figures 1A-D. Two illustrative patients (‘X’ and ‘Y’) both scheduled for subcutaneous apomorphine infusion are indicated, on the plots. Despite showing comparable levels of perceived severity in most of the problem domain, patients X and Y show very different expected outcomes within three of the four domains. Schematic views of profile scores across each of the PRO-APD items are shown in Figure 2 (severity) and Figure 3 (expectation). To illustrate this further the following brief cases vignettes are provided:

Patient X: 55 year old male, with a 9 year history of PD, receiving treatment with ropinirole 24 mg, and levodopa/carbidopa (100 mg/25 mg) 6 per day. His off-state MDS-UPDRS-3 score was 83. There was no evidence of cognitive impairment (ACE-R = 100) and only moderate levels of depression/ anxiety (HADS total 12). His perceived severity ratings indicated high levels of motor symptomatology. He reported a range of marked non-motor problems including sleep disturbance, bladder problems, pain and fatigue. Cognitive and mood complaints were mild, but significant impulsive behavior reported. Apart from self-care, aspects of occupation and social function were subjectively markedly affected. The patient expected the majority of the problem reported across all domains rated to be ‘very much improved’ by advanced treatment.

Patient Y: 56 year old male with a 13 year history of PD, receiving treatment with rotigotine 16 mg, levodopa/carbidopa (100 mg/25 mg) 18 per day, and levodopa/carbidopa controlled release (200 mg/50 mg) at night. His off-state MDS-UPDRS-3 score was 60. There was evidence of significant cognitive impairment (ACE-R = 73) and significant depression/anxiety (HADS total = 18). He reported significant motor symptoms including problems with swallowing and balance, plus fatigue, cognitive difficulties, and depression. With the exception of impact on socializing, however, his PD was not felt to have significant impact on other activities. Despite the range and severity of problems reported, he had only moderate expectations for the impact on treatment on the motor and non-motor symptoms, with no expectation of change for the cognitive or mood problems.

Across domains, the perceived severities of the motor problems were associated with the severity of non-motor problems (r = 0.66, p < 0.001) and social problems (r = 0.44, p < 0.05) but not the cognitive/psychological problems (r = 0.16, p > 0.05). No association was found between any of the other pairs of domains. For the expectation scores, associations were higher for all domain pairs (motor:non-motor, r = 0.72 , p < 0.001; motor:cognitive/psychological, r = 0.51, p < 0.05; motor:social, r = 0.65, p = 0.001; non-motor:cognitive/psychological, r = 0.44, p < 0.05; non-motor:social, r = 0.63, p < 0.01; cognitive/psychological:social, r = 0.59, p < 0.01).

The detailed associations between the perceived severity and expectation domain scores and total scores, with a range of clinical measures (UPDRS-III total score, NMSS totals score, ACE-R total score and HADS total score) are given in Table 3. Clinician rated motor severity was moderately associated with patient perceptions of motor problems but not with any other domain. Total NMSS score was associated with all domains, most strongly with the perceived severity of cognitive and psychological problems. The level of depression and anxiety as measured by the HADS was strongly associated with perceived severity of cognitive and psychological problems, with cognition function as measured objectively by the ACE-R being unrelated to any of the perceived severity scores. For expectation scores, UPDRS-III and NMSS were moderately associated with expected motor change. NMSS, HADS and ACE-R were all associated with greater expectation in the cognitive and psychological domain, with worse NMSS, greater depression/anxiety and better cognition associated with greater expected improvement.

**Table 3:** Correlations coefficients * between clinical variables and PRO-APD severity and expectation domain scores (N = 22)

	**UPDRS 3-total**	**NMSS total**	**HADS total**	**ACE-R total**
**Motor severity**	0.40	**0.49**^**a**^	0.31	0.15
**Motor expectations**	0.37	**0.46**^**a**^	0.24	0.36
**NMS severity**	0.15	0.40	0.27	0.17
**NMS expectations**	0.02	0.36	0.18	0.35
**Cognition/Psychological severity**	0.15	**0.62**^**b**^	**0.57**^**b**^	0.16
**Cognition/Psychological expectations**	0.14	**0.61**^**b**^	**0.58**^**b**^	**0.45**^**a**^
**Social severity**	0.26	**0.59**^**b**^	0.32	0.20
**Social expectations**	0.00	0.23	0.25	0.30
**Total severity**	0.31	**0.70**^**c**^	0.43	0.27
**Total expectations**	0.20	**0.49**^**a**^	0.34	**0.45**^**a**^

*Spearman rank correlation coefficient. UPDRS-3 = Revised Unified Parkinson’s Disease Rating Scale (Part 3); NMSS = Non Motor Symptom Scale; HADS = Hospital Anxiety and Depression Scale; ACE-R = Addenbrooke’s Cognitive Examination (Revised).

^a^p < 0.05; ^b^p < 0.01; ^c^p < 0.001. Unmarked ≥0.05. The values in bold are the ones with significant p values.

## Discussion

The aim of this pilot study was to design a multidimensional PRO assessment tool for PD patients scheduled for an invasive therapy, and then to assess its practicality in application and potential clinical utility at both group and individual level. The measure is novel in combining subjective perceptions of symptom difficulty with expectations of treatment outcome. Despite the small numbers of patients assessed, we have shown that such a scale can be pragmatic to use, even in a complex multimodal disease, such as PD, and that it is able to provide clinically meaningful information to supplement routine clinical assessment. This approach allowed us to determine for each individual a map of the patient’s perceptions of their disease and their expectation from treatment, and offer the potential to help manage patients in preparing them for therapy.

Traditionally, the measurement of improvement of parkinsonism by therapy has been by measuring motor signs and broad measures of outcome, such as health-related quality of life [13,14], The multiplicity of symptoms in PD means that motor scores are not sufficient to describe overall quality of life [3,9,15]. The impact of therapies on the other aspects of PD is generally less well understood and has been explored less than motor state [15-17]. This is in line with data from others and our group that suggests that standard PD severity scales are an insufficient measure of disease burden. Indeed, the “gold standard” Unified Parkinson’s Disease Rating Scale (UPDRS) has been modified to the “new UPDRS” (MDS-UPDRS) for this reason [8]. Despite an improvement as measured by standard scales, patients may describe being unhappy after DBS [16,18]. Conversely, patients may be very satisfied despite modest clinical gain. From our department, we have noted a patient whose main desire for treatment of her PD was the ability to “sit still and sew” [19]. Her degree of satisfaction following DBS (“very pleased”) was not matched by the minor improvement (< 10%) in the UPDRS score. It is debatable which outcome measure was the more clinically relevant. Further, the assessment of “success” from a therapy can become more complex if one were to extend analyses to include a more “complete” assessment of stage of life, which would include social dynamics including relationships/marriage [20]. Measuring treatment effects on one symptom (e.g. tremor), or set of symptoms (e.g. the ability to move), is insufficient to determine if a therapy is multi-dimensionally beneficial for a patient, cohort or population, unless it is known that it is that symptom (or set of symptoms) which is personally significant to the patient. For example, we have observed that despite “successful” DBS as determined by the patient, neurologist, neuropsychologist, neuropsychiatrist and family practitioner, a carer remained unhappy because the partner had “changed”, no longer needing such intensive support from the carer, leading to a mutually acceptable marriage breakdown. In this complex circumstance, should the treatment be considered “successful”? Should it be offered to similar patients?

The PRO concept informing the present study is a move away from assessments based on the clinical aim of “fixing” the symptom/sign, and towards selecting treatments that are best able to address a patient’s priorities across the range of PD-related problems and their impact. The concept is powerful and yet pragmatic, and is in line with trends that encourage patient inclusion in treatment decisions. This is a simple means of assessing the results of severity and expectation for individuals and its meaning to the patient [6]. Utilization of any PRO does not move away from the need to use standard severity scales, nor generic or specific quality of life scales. However, they can help clinicians and researchers, jointly with the patient, to understand better the impact of symptoms and proposed treatments (rather than the physiological effect of treatment on the sign). PRO-APD draws on three decades of experience in the assessment of other chronic multifactorial disorders (e.g. dementia [21], schizophrenia [22] and neurorehabilitation [23]). Most importantly, there is now guidance from both the FDA and European Medicines Agency for the use of PRO measures in drug development [24] and so one expects to see their increasing use over the next generation of treatments.

As a first step, we report here the preliminary use in assessing severity and expectation in a common disease, even if it is as complex as PD. A further study is required to validate this PRO-APD and demonstrate its utility, and for assessment of objective and subjective outcomes after therapy to determine whether “expected outcome” is matched to “measured outcome”, and if not what the impact of the discrepancy might be. With further extrapolation, the measure should be equally applicable to other adult or paediatric chronic movement disorders, like essential tremor and dystonia with adjustment where necessary of the problems assessed.

Unless a PRO is intended as a surrogate for traditional conventional measures (e.g. self-report version of a clinical scale for use in surveys), strong association are not necessarily expected or even necessary. In the present case, patients were asked to assess the severity of the PD-related problems and expectations of change. Given the difference in the various scales, item content and methods of assessment, we would expect only moderate associations between self-rated problem domain severity and standard scales. This was observed for motor problems (with MDS-UPDRS part 3, shown in Table 3), NMS problems (with NMSS, shown in Table 3) and psychological and cognitive problems with (HADS but not ACE-R, shown in Table 3). This latter result suggests that the Cognitive/Psychological domain may be more sensitive to measuring mood problems in the present sample. Subjective ratings of cognition have also typically been found to be unrelated to objective measurements across the age span [25].

At a group level, there was evidence for a robust association between the perceived severity of the different problems domains and the expectations for outcome, with all patients expecting improvement or, at worse, no change. We found that the associations between severity and expectations were strongest and most reliable for the motor and non-motor symptom domains, and less robust for the cognitive and psychological problems and weakest for the social and ADL domain (See Figure 1A-D). This may reflect the patient’s model of their disease and the degree to which they linked different problems to the underlying PD, and therefore amenability to treatment. For example, one can speculate that a patient who attributes memory problems to his/her age may have different expectations from a treatment targeting PD compared with a patient who sees the memory problems as an integral feature of the disease. Helping patients to understand the nature and extent of the full range of problems associated with their PD, and the potential impact of therapy, is likely to be important in helping them make informed choices and assess the possible impact of any future treatment.

Low mood might conceivably have had an impact on expectations, with depressed and anxious patients expecting a less favorable outcome. In practice, depression and anxiety as measured by the HADS was associated positively with degree of expected change, although the effect was significant only for the cognitive and psychological problems.

Clinical outcomes from advanced treatments such as DBS, Apo and IJL can produce dramatic improvements in some patients and for some symptoms, both motor and non motor. However, not all patients show a marked improvement and not all symptoms show similar responsiveness. An assumption that the most severe (and personally significant) problems are going to improve, and by the largest extent, raises the risk that patients may be dissatisfied with the outcome [18,26]. Identifying such misperceptions in advance offers the opportunity to provide the patient with the facts to inform and possibly adjust their expectations of treatment ahead of time. Although lack of accurate information may contribute to unrealistic expectations, individual characteristics (such as optimism) may also play an important role. Optimism is recognized as a dispositional trait that is largely independent of factors such as mood [27]. Evidence from patients with other physical conditions suggest that low levels of optimism is associated with poor outcome, perhaps because patients fail to engage with treatment or to be proactive in coping with the challenges of their condition. However, high levels of optimism may also be unhelpful if they set up expectation that cannot be met or encourage a passive ‘everything will be alright’ attitude. Practically, moderate (rather than excessively high or low) levels of optimism may be the best for outcome in chronic disease such as multiple sclerosis and Parkinson’s disease [28] and something that clinicians can encourage when discussing potential treatments.

The two cases described in this study were chosen to illustrate somewhat polarized expectations with case X having extreme levels of positive expectation for the large majority of problems experienced (which may not have been achievable), while case Y had lower expectations for some and no positive expectations for other problems. Both patients might benefit from a detailed discussion about what the planned treatment can and cannot reasonably offer. Patient Y was more depressed and had a significant degree of cognitive impairment that may have biased their judgment. However, a more pessimistic and limited set of expectations is not necessarily bad and may even be closer to the likely clinical reality. A danger of low expectation is when they cause a patient to decline available treatment options.

We note several important limitations to our study. Our study is a small pilot and further research is required to demonstrate the scale validity and utility. We appreciate the current lack of accepted consistency in meaning of the term “holistic”, and of uniform guidelines on the specific selection of one treatment modality over another, although some guidance exists, e.g. significant cognitive impairment may exclude DBS, but not always exclude IJL or Apo [4]. None-the-less, this is a pragmatic first step which exemplifies the benefits of a PRO approach, and which if developed further could help guide individual patients to be offered and accept individual therapies.

In conclusion, a PRO approach encompasses a patient-driven view of care. We present a pilot study using PRO-APD as a demonstration to provide a simple and pragmatic assessment of the severity and expectation of treatment, and which is in line with increased patient participation in their management. Patients opting for invasive therapy for PD have moderately high multi-domain symptom load, and also expect substantial improvements in multiple domains. These need to be considered when assessing patients for therapy, so that individual expectations can be realistic, since unexplored expectations are more likely to lead to overall dissatisfaction following therapy. Our findings are preliminary. Further validation could lead to the use of PRO-APD as an adjunct to existing scales in clinical management.

## Appendix

Items from PRO-APD

Motor domain

1. Tremor. Has tremor/shaking affected any part of your body?

2. Stiffness or slowness moving. Have you found it difficult to move or carry out everyday activities because of stiffness in your muscles or slowness moving?

3. Off Periods. Times when your medication does not seem to work properly of where the effects wear off before the next tablet is due. Do you have times when you remain slow, stiff or shaking even if you have taken your medication?

4. Dyskinesia. Jerky movements in any part of your body that you cannot stop? I don’t mean tremor. Have you had times when a part of your body has been moving more than usual without you trying to move it?

5. Freezing. Times when you suddenly freeze and cannot move? Have you felt as if your feet are stuck to the floor?

6. Dystonia. Have you had problems with muscle spasms, abnormal or distorted posturing or painful spasms of muscles?

7. Speech. Have you had problems with your speech, with difficulty in speaking loudly or clearly? Have people had difficulty understanding you when you talk?

8. Balance / Falls. Have you fallen or felt off balance and thought you might fall? Have you felt unsteady when turning around or when leaning over?

NMS domain

9. Swallowing. Have you had problems swallowing liquids or solids? Have you found yourself dribbling while drinking or choking while eating?

10. Sleep. Do you have problems with sleep, like falling asleep at night or staying asleep? Is your sleep disturbed, for example by unpleasant dreams?

11. Bowels. Do you have problems with your bowels like constipation, loose stools? Do you need laxatives?

12. Bladder. Do you have problems like urgency, incontinence or need to go to the toilet too frequently at night? Do you have difficulty holding urine?

13. Pain. Do you suffer from any unexplained pain other than conditions like arthritis or muscular injury? Do you have pain or other unpleasant feeling in any part of your body?

14. Fatigue. Have you experienced extreme tiredness or lack of energy during the day? I don’t just mean feeling sleepy. Do you feel fatigued even though you have not been active?

15. Sexual function. Have you had loss of interest in sex? Have you had difficulty with sex if you have tried? Have you been unable to have sex compared to how you would like?

Cognition and psychological domain

16. Concentration. Do you have problems concentrating, for example during a conversation or while reading a book or newspaper? Do you find it difficult if you are given too much information at once?

17. Memory. Do you have problems remembering things? Do you forget where you have left things or forget things that you meant to do? Do other people have to remind you?

18. Impulsive behaviour. Do you do any of the following: do you gamble more than you can afford (wait for reply). Do you spend a lot of money shopping? Are you extremely interested in sex? Do you find it hard to throw things away?

19. Hallucinations/Psychosis. Have you heard or seen or smelled anything that you knew was not really there, or other people have told you were not there? Have your senses played tricks on you?

20. Mood. Do you feel low in mood, sad, hopeless or unable to enjoy things? Have you felt blue or ‘empty’

21. Anxiety. Have you been troubled by worries that you cannot get rid of? Have you felt frightened or panicky? Have you felt tense and nervous?

22. Apathy. Do you have difficulty motivating yourself to do the things you need to do? Do you need prompting and encouragement? Do you spend much time sitting doing nothing?

Social and ADL domain

23. Self-care. Do you have difficulty managing your day-to-day activities for caring after yourself? Do you have problems washing, dressing and eating your food?

24. Work. Do you still work? If so, do you have difficulty doing your job because of your Parkinson’s disease?

25. Leisure/Hobbies. Do you have difficulty with any of your leisure activities or hobbies? Have you recently had to stop doing things that you enjoy?

Socialising. Do you find it difficult to socialize, either in your home or outside? Do you find social situations difficult?

## Competing interests

The authors declare that they have no competing interests. Authors RGB and KRC receive salary support from the National Institute for Health Research (NIHR) Mental Health Biomedical Research Centre and Dementia Research Unit at South London and Maudsley NHS Foundation Trust and King’s College London. The views expressed are those of the authors and not necessarily those of the NHS, the NIHR or the Department of Health.

## Authors’ contributions

1. Research project: A. Conception, B. Organisation, C. Execution; 2. Statistical Analysis: A. Design, B. Execution, C. Review and Critique; 3. A. Writing of the first draft, B. Review and Critique, PR: 1A, 1B, 2A, 2B, 3A, 3B. P MM: 1A, 1B, 2A, 2B, 3B. RG Brown (RGB): 1A, 1B, 2A,2B, 3B. K Ray Chaudhuri (KRC): 1A, 1B, 2A, 2B, 3B. J-PL: 1B, 2A, 3B. R Selway: 1B, 2A, 3B. IF: 1B, 2A, 3B. KA: 1A, 1B, 3B. MS: 1A, 1B, 2A, 2B, 3A, 3B. All authors read and approved the final manuscript.
